# The role of delayed aortic surgery in type A aortic dissection and mesenteric ischemia: a systematic review and meta-analysis

**DOI:** 10.1186/s13019-023-02341-y

**Published:** 2023-08-18

**Authors:** Aditya Eranki, Ashley R Wilson-Smith, Michael L Williams, Aashray Gupta, Campbell Flynn, Jim Iliopoulos, Con Manganas

**Affiliations:** 1 Department of Cardiothoracic Surgery, Royal Prince Alfred Hospital, Sydney, Australia; 2 John Hunter Hospital, New Lambton Heights, Newcastle, Australia; 3 The Collaborative Research Group (CORE), Sydney, Australia; 4 The University of Sydney, Sydney, Australia; 5 Department of Cardiothoracic Surgery, Gold Coast University Hospital, Gold Coast, Australia; 6 University of Adelaide, Adelaide, Australia; 7 Department of Cardiothoracic Surgery, St George Hospital, Kograh, Sydney, 2217 Australia; 8 Department of Vascular Surgery, St George Hospital, Kograh, Sydney, Australia

**Keywords:** Acute type a aortic dissection, Mesenteric ischemia, Malperfusion, Management, Mesenteric malperfusion, Outcomes

## Abstract

**Introduction:**

Approximately one third of patients with Acute Type A Aortic Dissection (ATAAD) present with pre-operative malperfusion syndromes (MPS). Of these, mesenteric malperfusion represents the greatest risk to patients with respect to increased short-term mortality. In select patients, it may be feasible to offer a staged approach by treating the mesenteric malperfusion first, optimizing the patient in the intensive care setting and then, following with a central aortic repair. The aim of this systematic review is to summarize cohort studies assessing the role of pre-operative interventions for mesenteric malperfusion.

**Methods:**

An electronic literature search of five databases was performed to identify all relevant studies providing studies examining short-term mortality on patients who underwent either endovascular or open revascularisation of mesenteric ischemia prior to central aortic repair. The primary outcome was all-cause, short-term mortality. Secondary outcomes were comparative mortality between a delayed repair vs. aortic repair first strategy, rates of postoperative laparotomy, bowel resection, and mortality following delayed aortic repair.

**Results:**

The search strategy identified 8 studies qualifying for inclusion, with a total of 180 patients who underwent delayed aortic surgery in the setting of mesenteric MPS. The weighted short-term mortality following a mesenteric revascularisation first, delayed aortic surgery strategy was 22.5%. This strategy was also associated with a significantly lower mortality than a central repair first strategy (OR 0.07, 95% CI 0.02–0.27), and a significantly lower rate of postoperative laparotomy/bowel resection (OR 0.05, 95% CI 0.02–0.14). If patients survive to receive central repair, the weighted short-term mortality postoperatively is low (2.1%).

**Conclusion:**

A summary of this evidence reveals a lower short-term mortality in hemodynamically stable patients with mesenteric malperfusion, along with a reduction in postoperative laparotomy/bowel resections. Of those patients who survive to receive central repair, short-term mortality remains very low in the select group of hemodynamically stable patients. Further high-quality studies with randomized or propensity matched data are required to verify these results.

**Supplementary Information:**

The online version contains supplementary material available at 10.1186/s13019-023-02341-y.

## Introduction

Acute type A aortic dissection (ATAAD) is a cardiothoracic emergency necessitating emergent surgical management, being associated with a short-term mortality ranging from 15 to 30% [1, 2]. Presentations for this pathology range in a spectrum from hemodynamically stable patients to those in cardiogenic shock and aortic rupture. Approximately one third of patients with ATAAD present with pre-operative end organ malperfusion syndromes (MPS) [3, 4]. These patients are at higher risk of mortality post-operatively. Of these, mesenteric malperfusion often represents the greatest risk to patients with a short-term mortality of 30% and a late mortality that is twice as high as patients without malperfusion syndromes [5, 6]. These patients are likely to have long postoperative courses in hospital requiring multiple postoperative procedures all resulting in greater morbidity [6].

Patients with malperfusion syndromes experience an inflammatory cascade stemming from end organ ischemia, which is an additional barrier to operative success [7]. Free radicals and inflammatory cytokines perpetuate end organ injury, resulting in multiorgan failure and mortality despite a surviving aortic repair [8]. Moreover, MPS due to static obstruction of branch vessels (such as thrombosis) may not be resolved by aortic repair alone, resulting in prolonged postoperative ischemia [9]. To date, the management strategy of MPS and ATAAD remains tenuous. There is evidence supporting favourable outcomes arising from immediate aortic surgery in the setting of malperfusion [10–13]. Subgroup analysis within these studies, however, demonstrate a significantly higher mortality rate in the group of patients presenting with mesenteric malperfusion [11, 13]. These studies advocate for a different treatment paradigm in these patients, highlighting the lethal nature of this condition [13]. In select patients, it may be feasible to offer a staged approach by treating the malperfusion first, optimizing the patient in the intensive care setting and then, following with a central aortic repair [14]. The majority of evidence reporting delayed central repair in the setting of mesenteric ischemia is in the form of case reports and small case series [14]. Larger institutional studies have been published recently reporting the role of endovascular and open repair of mesenteric malperfusion prior to central aortic repair, however these are heterogenous and encompass a broad range of repair strategies [15].

The aim of this systematic review is to summarize cohort studies assessing the role of pre-operative interventions for mesenteric malperfusion. The primary outcome of the review is to report the short-term mortality in the cohort of patients undergoing delayed surgery in the setting of mesenteric malperfusion. Secondary outcomes assessed were the comparative short-term mortality between delayed and immediate central repair cohorts, comparative rates of laparotomy with or without bowel resection and short-mortality of those patients who survived to receive aortic repair in the delayed cohort.

## Methods

### Literature search strategy

This trial was written in accordance with Preferred Reporting Items for Systematic Reviews and Meta Analysis (PRISMA) recommendations [16]. An electronic literature search of five databases: Ovid MEDLINE, EMBASE, Cochrane Central Register of Controlled Trials (CCRCT), Cochrane Database of Systematic Reviews (CDSR), and Database of Abstracts of Review of Effectiveness (DARE), was performed from time of inception to August 2022. The search strategy contained a combination of keywords and Medical Subject Headings (MeSH) including “Type A aortic dissection” AND “management” AND “((mesenteric ischemia) OR (malperfusion) OR (ischemia))”. Two reviewers (A.E, A.W.S) assessed the eligibility of the selected papers. Discrepancies between full text reviews were adjudicated by a third author (C.D.F). References of included articles were also crosschecked to see if additional studies could be identified.

### Inclusion and exclusion criteria

Studies were included if (1) patients had a Type A Aortic Dissection and mesenteric malperfusion (2) included patients who underwent either endovascular or open revascularisation of mesenteric ischemia prior to central aortic repair (3) reported mortality of this cohort. Studies were excluded if they did not delineate if patients had a Type A or Type B Aortic Dissection, did not delineate mortality in patients with mesenteric malperfusion from other forms of malperfusion, published in a language other than English or if full text article was not available.

### Data extraction and outcomes of interest

Treatment strategy, lactate and base excess, presence of other malperfusion syndromes, operative strategy and time to definitive repair were collected and entered into pre-defined tables. Outcomes including overall in hospital or 30-day mortality, rates of laparotomy and bowel resection, rates of aortic rupture (whilst waiting definitive surgery) and death following definitive aortic surgery in the delayed cohort was collected. If patients presented with multiple forms of malperfusion, the IPD was analysed where available and only patients whose primary source of malperfusion was mesenteric in nature were included. Patients whose primary issue was another source of malperfusion (cerebral, limb, cardiac) but who also had concurrent visceral malperfusion were excluded. Results were collected by two independent reviewers (AE and AWS). The primary outcome was all cause short-term mortality following a mesenteric reperfusion first and subsequent aortic surgery strategy (termed “delayed” cohort). The secondary outcomes were comparative mortality in patients undergoing a delayed strategy vs. central repair first strategy (termed the “control cohort”) in the setting of MI, rates of laparotomy (with or without bowel resection) in both cohorts and postoperative mortality following central repair in the delayed cohort, excluding those patients who died awaiting definitive aortic surgery.

### Statistical analysis

Statistical analysis was carried out using Stata® (Version 17.0, StataCorp, Texas, USA). For prevalence, a meta-analysis of proportions was performed using the *metaprop* function, with a Freeman-Tukey arcsine transformation. A random effects model was utilised to account for varied study design, year of publication, centre protocol and population. Results were expressed as forest plots were appropriate, with cumulative proportion expressed as a single percentage. For binary results comparing two cohorts the *meta esize* function was utilised, with effect size expressed as Peto’s log odds-ratio as numbers reported were generally small. Statistical significance was signified as P < 0.05. Heterogeneity was assessed using the I^2^ test statistic. Low heterogeneity was denoted by I^2^ < 50%, moderate heterogeneity by I^2^ 50–74%, and high heterogeneity by I^2^ > 75%.

### Study quality appraisal and publication bias

Study quality was assessed using the Risk of Bias in Non-Randomized studies of intervention (ROBINS-I) tool, outlined by *Sterne et al.* [17]. Publication bias was assessed through visual inspection of funnel plots and Eggers test in Stata®. A trim and fill analysis was performed in instances of publication bias. An influential study analysis with adjusted effect sizes computed after the omission of each study. In order to assess the impact of study age (recruitment year) on mortality, a meta regression was performed comparing year of publication to mortality in the delayed cohort, utilising a random effects model. A coefficient *n* was calculated to assess correlation and P value, with P < 0.05 denoting significance.

## Results

### Baseline study characteristics

A total of 710 abstracts were reviewed after 144 duplicates were removed. A further search of references of included articles provided an additional 4 studies, and 21 articles were chosen for full text review. Of these 8 studies were included [7, 18–24] (supplementary Fig. 1). Study dates ranged from 1992 to 2017. All eight studies reported mortality in patients undergoing delayed surgery for ATAAD, with re-establishment of visceral malperfusion first. The delayed cohort contained a total of 180 patients. six studies provided comparative cohorts of patients undergoing central repair first followed by repair of malperfusion, and included 45 patients. Preoperative biochemistry in the delayed cohort was reported by three studies and the mean lactate ranged from 3.3 to 7 [18, 19, 22]. Operative strategy was reported in most studies. In the delayed cohorts, patients underwent angiography and fenestration in four studies [7,20,22.23], TEVAR was performed in one study and laparotomy, bypass or atom tube placement in two studies [19, 21]. Three studies report using a hybrid operating theatre [18, 23, 24], with one study performing central repair immediately after mesenteric reperfusion. Time to central repair varied significantly across studies. One study performed central repair uniformly 24 h after mesenteric reperfusion, whereas others waited for biochemistry to normalise and the patient to recover, resulting a delay up to 3 weeks [7]. Two studies did not report the time to definitive repair [19, 24]. Inclusion criteria for the delayed cohort also varied significantly. The majority of studies opted to delay surgery central repair in patients with mesenteric malperfusion who were hemodynamically stable. Two studies had unclear inclusion criteria for patients, stating a change in treatment paradigm over time to delayed surgery in the setting of mesenteric perfusion [7, 21]. One study was subject to further conditions such as operator preference, availability of the hybrid OR and age parameters [23]. These results are summarised in Table 1.

**Table 1:** Study characteristics

Authors	Yrs	Design	Study Cohort	“Delayed” cohort	Comparison cohort “control”	Delayed cohort inclusion criteria	Control inclusion criteria.	Definition Malperfusion	Preoperative biochemistry (delayed)	Delayed cohort strategy	Control cohort strategy	Hybrid theatre?	Time to definitive (aortic) repair
Leshnower et al. [18]	2003 2017	Retrospective	ATAAD with MPS	ATAAD and MM	Central repair first in setting of MM	Carefully selected patients. CI to haemodynamic instability.	Haemodynamic instability /rupture	CT Cross sectional evidence of ischemia along with symptoms	Reported as individual patient data	13 TEVAR 3 ax-bifemoral bypass	10 hemiarch 2 root replacements	Yes	24 H after reperfusion
Yamashiro et al. [19]	2000 2014	Retrospective	ATAAD and visceral malperfusion	ATAAD and MM	Central repair first in setting of MM	Carefully selected patients. CI to haemodynamic instability,	Haemodynamic instability /rupture	CT Cross sectional evidence of ischemia along with symptoms	BE: −2.8 ± 1.2 Lactate 3.3 ± 1.2 a	Emergency Laparotomy/bypass before central repair relook laparotomy post central repair	Not Specified	No	Not reported
Patel H et al. [20]	1997 2007	Retrospective	Patients with ATAAD malperfusion (all)	ATAAD and MM (subgroup)	No - Not specified for MM p	Carefully selected patients. CI to haemodynamic instability,	N/A	CT Cross sectional evidence of ischemia along with symptoms	Not reported	Angiography with percutaneous fenestration and aortic true lumen stenting with or without branch vessel stenting	N/A	No	median 4 days after reperfusion
Uchida et al. [21]	2006 2016	Retrospective	Patients with ATAAD and CT or clinical evidence of malperfusion (all types)	ATAAD and MM	Central repair first in setting of MM	Unclear – potentially change in protocol over later to contemporary time periods	Unclear – potentially change in protocol over later to contemporary time periods	CT Cross sectional evidence of ischemia along with symptoms	Not reported	Laparotomy and atom tube into SMA	Not Specified	No	Immediately after reperfusion
Deeb et al. [7]	1992 1996	Retrospective	Patients with ATAAD and CT or clinical evidence of malperfusion (all types)	ATAAD and MM	Central repair first in setting of MM	Unclear – potentially change in protocol over later to contemporary time periods	Unclear – potentially change in protocol over later to contemporary time periods	visceral: pain associated with physical findings compatible with an acute abdomen and associated abnormal laboratory findings.	Not reported	Fenestration of the aortic dissection flap accompanied by the stenting of compromised branch vessel	Root = 6, Hemiarch = 2, Ascending aorta = 1, total arch = 2	No	Median (days) 23 (IQR 2–57) after reperfusion
Yang et al. [22]	1996 2017	Retrospective	ATAAD with mesenteric malperfusion	ATAAD with MM	No (single arm study)	Carefully selected patients. CI to haemodynamic instability,	N/A	CT Cross sectional evidence of ischemia along with symptoms	7 ± 8.5 (max serum lactate mean)	IR, endovascular fenestration ± SMA stenting or suction thrombectomy if static obstruction 10 patients required further laparotomy and bowel resection.	Root replacement 14, Root repair 28, Arch 27, FET 2.	No	Median (days) 6 (IQR 2–19)
Tsagakis et al. [23]	2004 2011	Retrospective	ATAAD and malperfusion (All)	ATAAD with MPS	Yes – Central repair first in the setting of MM (subgroup)	Carefully selected patients. CI to haemodynamic instability,	Rupture/CPR and other reasons: Age less than 50 years (6/34), cardiogenic shock (17/34) Tamponade (5/34) previous coronary angiograms (2/34) unavailability of the Hybrid OR (4/34)	CT Cross sectional evidence of ischemia along with symptoms	Not reported	All endovascular: four fenestrations, six splitting of the true limen,	Not Specified	Yes	Immediately after
Sugiyama et al. [24]	2017–2019	Retrospective	ATAAD with mesenteric malperfusion	ATAAD with MPS	Central repair first in setting of MM	Carefully selected patients. CI to haemodynamic instability,	Haemodynamic instability /rupture	CT Cross sectional evidence of ischemia along with symptoms	Not reported	Not reported	Hemiarch = 2, total arch = 2	Yes	Not reported

### Primary outcome

**Fig. 1:**
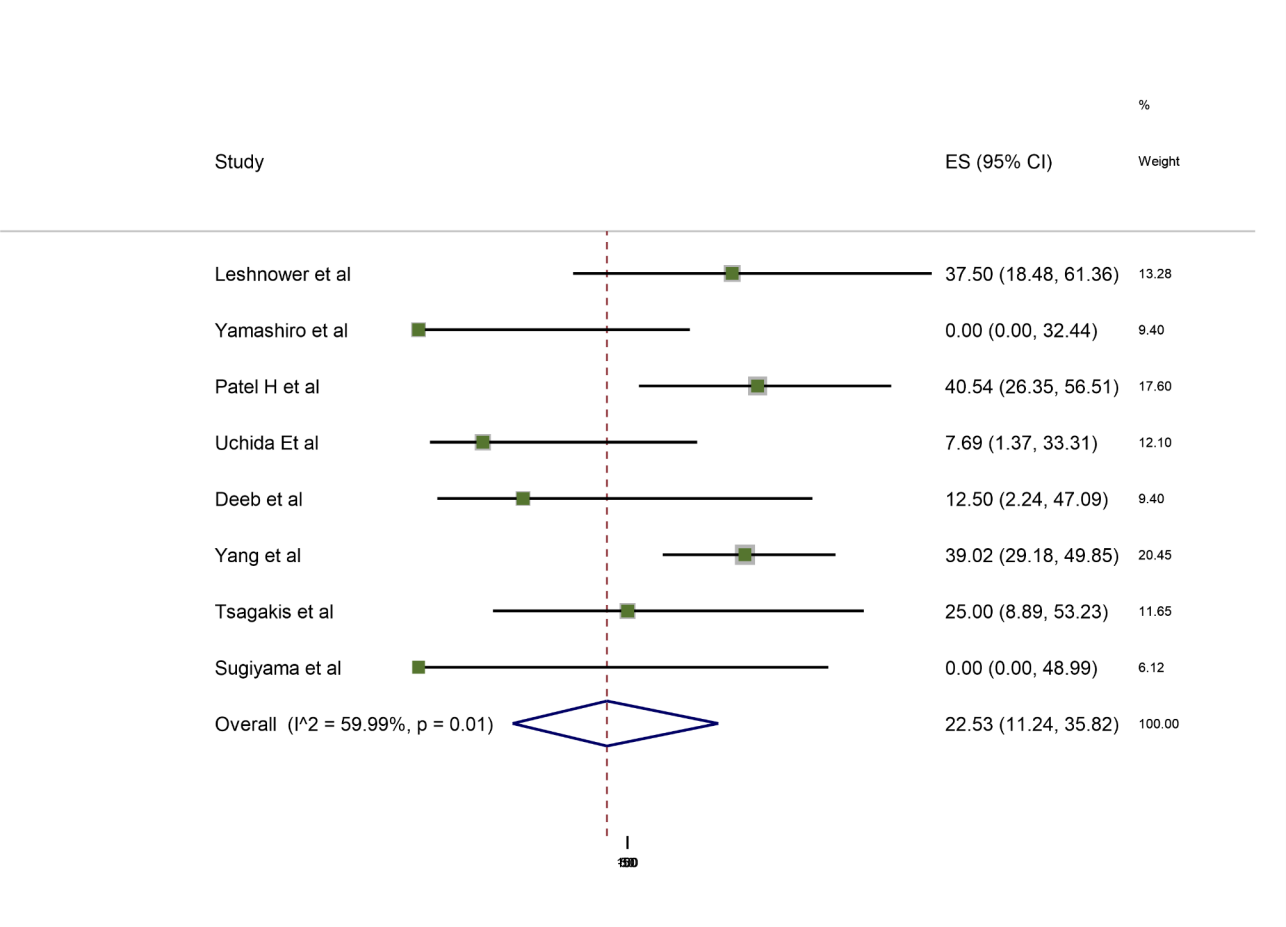
Overall 30-day mortality delayed cohort

All eight studies reported the primary outcome, short-term mortality following a mesenteric reperfusion first and subsequent aortic surgery (“delayed” cohort). There were 58 deaths overall out of 180 patients (mortality rate of 32%). A meta-analysis of proportions demonstrated a weighted short-term mortality of 22.5% (95% CI 11.2–35.8%) taking onto account the weight of each study and associated with moderate heterogeneity (I^2^ = 60%) (Fig. 1). On visual inspection of the forest plot, *Yang et al.* was identified as potentially an influential study (supplementary Fig. 2). The subsequent omission of this study did not affect heterogeneity or effect size significantly with a weighted short-term mortality of 18.1% (I^2^ = 56.6% ).

### Secondary outcomes

**Fig. 2:**
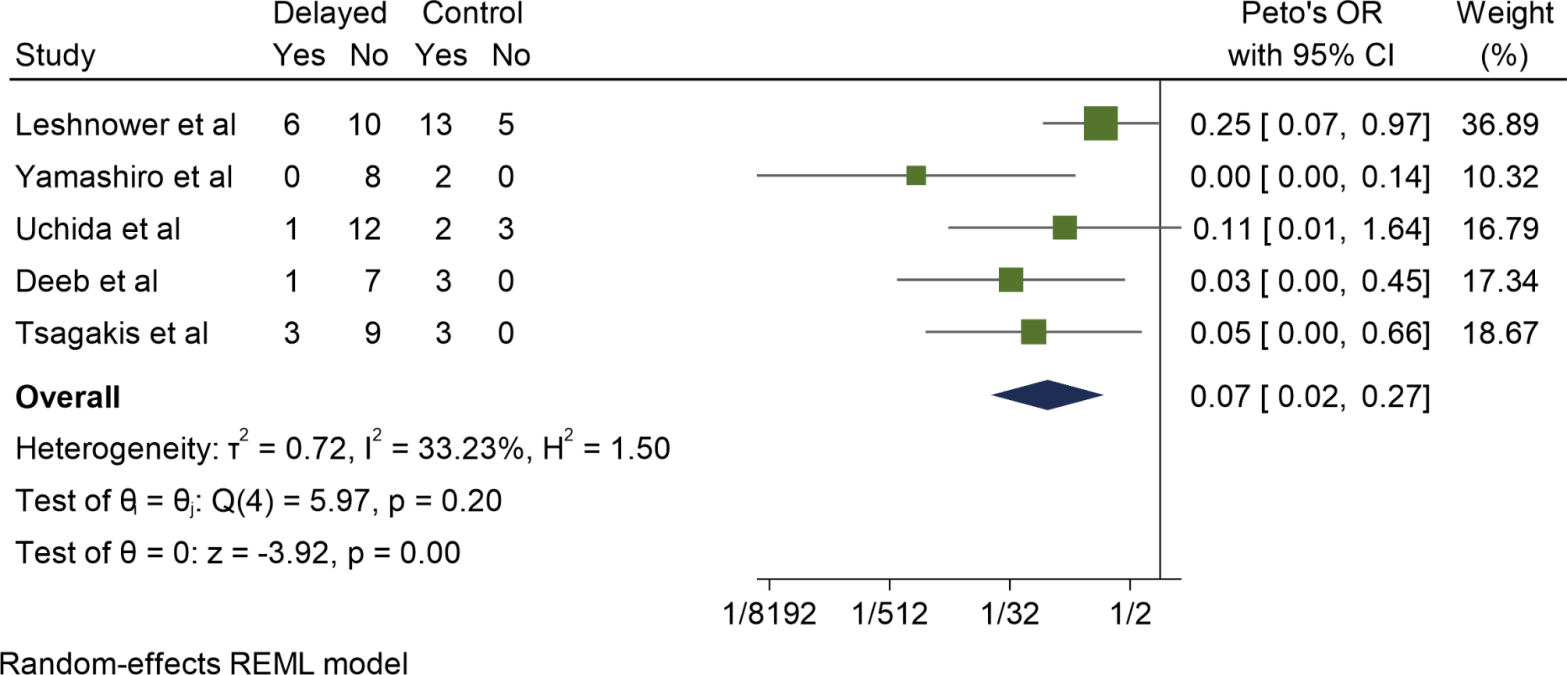
Comparative 30-day mortality between delayed and control cohorts

**Fig. 3:**
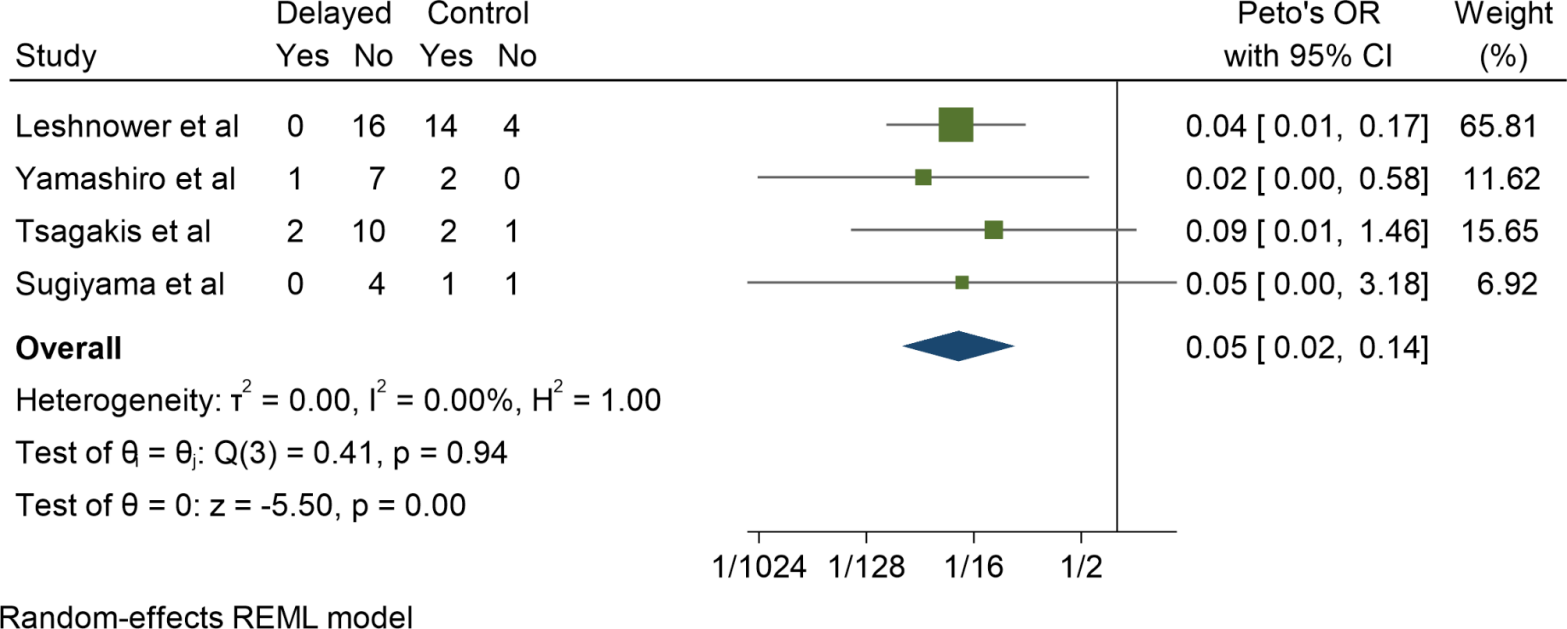
Postoperative laparotomy/bowel resection delayed vs. control cohorts

Five studies compared mortality between delayed and control cohorts in the setting MI. Mesenteric reperfusion first followed by aortic surgery was significantly associated with lower mortality, with a Peto’s OR of 0.07 (95% CI 0.02–0.27, P < 0.01). This result was associated with low heterogeneity (I^2^ = 33%) (Fig. 2**)**. Of note, four of the five studies demonstrated a significantly lower mortality in the delayed cohort. Delayed surgery also conferred a significantly lower rate of post-operative laparotomy when compared to the control cohort, with a Peto’s OR of 0.05 (95%CI 0.02–0.14, P < 0.01). This result was associated with low heterogeneity also (I^2^ = 0) (Fig. 3).

**Fig. 4:**
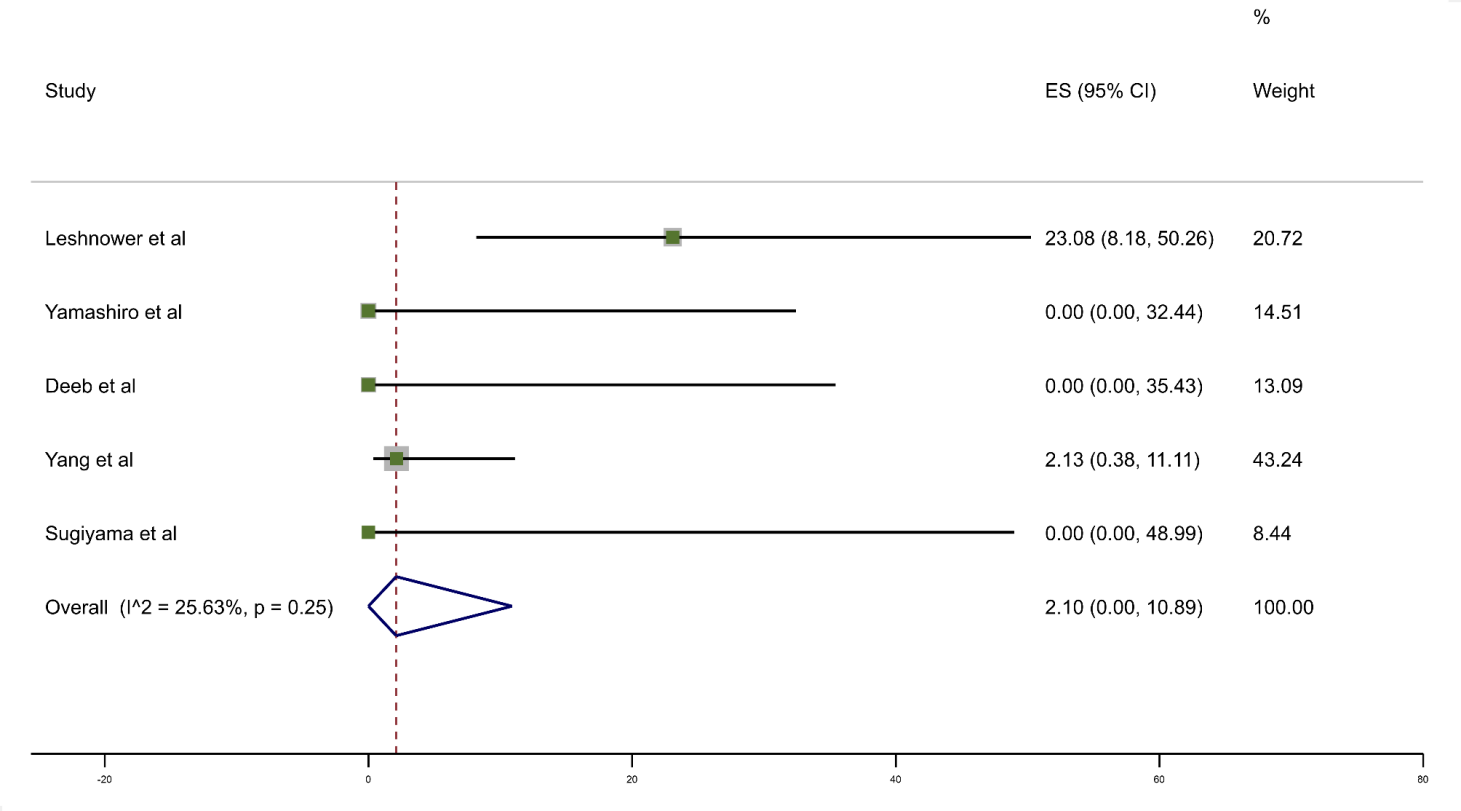
30-day mortality in those patients who survived to undergo aortic repair

Five studies assessed mortality following central repair in patients who survived the initial mesenteric reperfusion. The weighted mortality was 2.10% (95% CI 0–10.89); a total of 4 patients (Fig. 4**)**. Of note, three studies reported no deaths following aortic surgery however these studies had low patient numbers. In these five studies, a total of 79 patients underwent aortic surgery. Thirty-five patients died prior to definitive surgery, with 13 of these (37%) as a result of acute aortic events. These results are summarised in Table 2.

**Table 2:** Outcome data

Authors	# Delayed Cohort	# Control Cohort	# patients survive to central repair (delayed cohort)	Post-op laparotomy/bowel resection delayed cohort	Post-op laparotomy/bowel resection control cohort	# patients died before central repair (delayed cohort)	# of these patients who died of aortic rupture	# patients who died after central repair (delayed cohort)	Overall short-term mortality delayed	Overall short-term mortality control
Leshnower et al. [18]	16	18	13 (81%)	0 (0%)	14 (78%)	3 (19%)	1	3 (23%)	6 (37.5%)	13 (72%)
Yamashiro et al. [19]	8	2	8 (100%)	1 (12.5%)	2 (100%)	0 (0%)	0	0 (0%)	0 (0%)	2 (100%)
Patel H et al. [20]	37								15 (41%)	
Uchida et al. [21]	13	5							1 (8%)	2 (40%)
Deeb et al. [7]	8	3	7 (87.5%)			1 (12.5%)	1	0 (0%)	1 (12.5)	3 (100%)
Yang et al. [22]	82		47 (57%)	9 (11%)		31 (38%)	11	1 (2%)	32 (39%)	
Tsagakis et al. [23]	12	3		2 (17%)	2 (67%)				3 (25%)	3 (100%)
Sugiyama et al. [24]	4	2	4 (100%)	0 (0%)	1 (50%)	0 (0%)	0	0 (0%)	0 (0%)	0 (0%)

### Study quality and assessment of bias

**Fig. 5:**
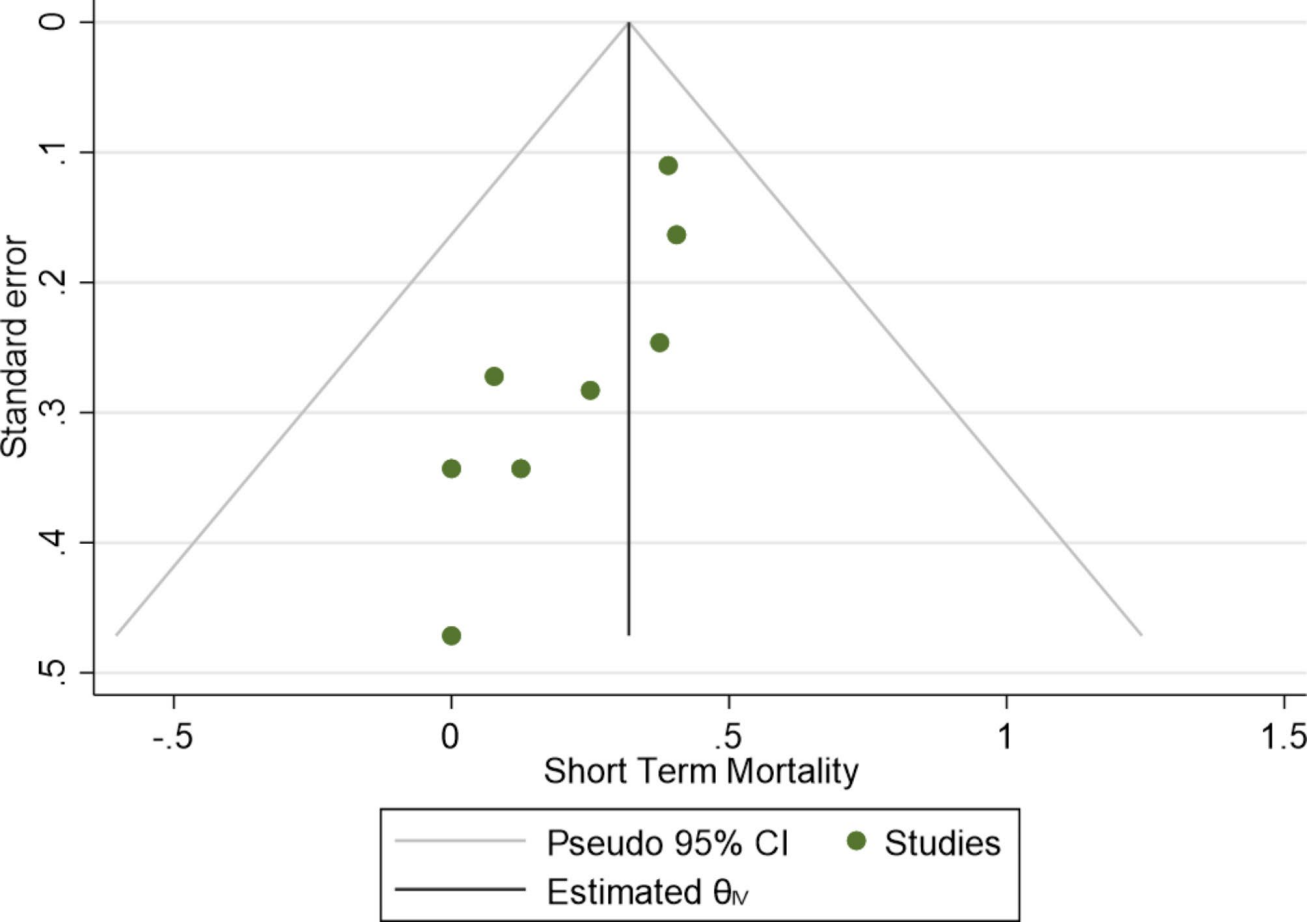
Funnel plot of overall short term mortality (delayed)

The risk of bias ranged from serious to moderate, with two studies having a moderate risk of bias and six studies demonstrating a serious risk of bias (Supplementary Fig. 3). A funnel plot was conducted demonstrating a degree of asymmetry, however Eggers test was performed and was not statistically significant (P = 0.12) (Fig. 5). Meta regression of recruitment year and effect size (mortality) was performed, and found no significant relationship between the two variables (P = 0.31) (Supplementary Fig. 4).

## Discussion

This systematic review demonstrates a few key findings:

- Weighted short-term mortality of 22.5% following a delayed strategy of mesenteric reperfusion first followed by central repair.
- A reperfusion first, delayed central repair strategy is associated with significantly lower mortality than a central repair first strategy (OR 0.07).
- The delayed cohorts demonstrated a significantly lower rate of postoperative laparotomy/bowel resection (OR 0.05).
- If patients survive to receive central repair, the weighted short-term mortality postoperatively is low (2.1%).

The classic management of ATAAD is to close the proximal entry tear, establishing true lumen perfusion. However, this may prolong the ischemic time of end organs, thus affecting their viability. The inflammatory effect of cardiopulmonary bypass, deep hypothermic arrest and the subsequent reperfusion injury further predisposes these patients to the release of inflammatory mediators causing severe multiorgan failure [7]. In the setting of mesenteric malperfusion, studies have reported paradoxical treatment paradigms, with an emphasis on addressing branch vessel obstruction first followed by aortic repair only when the malperfusion syndrome is resolved [15, 22, 25]. The pattern of branch vessel obstruction guides management [26]. Dynamic compression occurs secondary to the motion of the intimal flap within the aortic lumen as a result from haemodynamic forces [27]. As the compression is dependent on blood pressure differential between the true and false lumen, this can be managed as a first line with reduction in blood pressure medication and if this fails, intervention such as fenestration is feasible. A static obstruction occurs when the dissection of the branch vessel causes obstruction, most often due to thrombus obstructing the true lumen [27]. This requires interventional correction such as a thrombectomy, stenting and in some instances, open branch vessel bypass. The patient then is stabilised and monitored in the intensive care setting, followed by definitive aortic surgery once the malperfusion syndrome resolves [20].

We report a weighted short-term mortality rate of 22.5% percent following delayed surgery in ATAAD with mesenteric malperfusion. The moderate heterogeneity is reflective of different protocols, time- periods and treatment paradigms adopted by each study. This result is similar to previously published results. A recently published systematic review and meta-analysis *Xu et al.* demonstrated promising results, with 9 deaths out of 42 patients (21.4%) in the delayed cohort and a significantly lower rate of postoperative laparotomy [28]. This study was limited by the inclusion of double arm studies only and the exclusion of others [28]. We opted to include single arm studies providing an additional 138 patients in the delayed cohort. In addition, we assessed the outcome of patients who survived to receive central repair, hypothesising that once patients survive the initial malperfusion insult and undergo aortic surgery, survival is comparable to patients without malperfusion preoperatively.

The secondary outcomes demonstrate three key findings. Firstly, there was a significantly lower short-term mortality in patients undergoing a revascularisation first, delayed aortic surgery in the setting of mesenteric malperfusion when compared to a central repair first strategy (OR 0.07). This result was also reflected in previous systematic reviews [28]. There are a number of factors leading to this. Firstly, ischemic bowel causes a significant metabolic acidosis and release of inflammatory mediators, causing multiorgan failure unless corrected in a timely manner [18]. Patients with intractable bowel ischemia in the delayed cohort were denied further central repair, and only patients who were ‘well enough’ progressed to surgery, potentially representing a selection bias [18, 28]. Furthermore, six out of eight studies selected patients for the delayed cohort based on haemodynamic stability, whereas unstable patients went directly for central repair and formed the ‘’control’’ cohorts. These patients are inherently more complex, unwell and have a higher postoperative mortality. Future studies, either randomised in design or incorporating propensity matching may further elucidate whether delayed surgery is associated with improved short-term mortality.

Secondly, a mesenteric reperfusion first, delayed central repair strategy is also associated with significantly lower rate of postoperative laparotomy (with or without bowel resection) than a central repair first strategy. *Xu et al.* published a similar result, with the delayed cohort associated with less mesenteric complications (OR 0.15, P < 0.01) [28]. *Yang et al.* noted that reperfusion of the superior mesenteric artery (SMA) with fenestration can be done in 30 min, whereas the time taken during aortic, surgery, cardiopulmonary bypass and hypothermic arrest may result in intractable bowel ischemia due to prolonged vasoconstriction, thus rendering bowel non-viable [22].

Thirdly, we report a low mortality in patients who survived to undergo aortic surgery, with a short-term mortality of 2.10% following definitive aortic surgery. Of note however, 35 patients (19%) died waiting for definitive surgery, approximately one-third of these due to aortic rupture. The majority of patients who died as a result of rupture are accounted for by *Yang et al.*, and occurred during the first decade of the study where impulse control therapy was less commonly adhered to [22]. Therefore, death as a result of aortic rupture was uncommon. The most common cause of death whilst awaiting definitive surgery was multi organ failure as a result of significant ischemic time [18]. Furthermore, it has been demonstrated that the survival curves of patients with malperfusion who eventually proceeded to operative repair are similar to those who underwent surgery for uncomplicated ATAAD [20, 22]. This result is not surprising as this the goal of treating patients with ATAAD with mesenteric malperfusion; whereby a delayed strategy promotes resolution of malperfusion prior to central repair, essential converting a malperfusion patient to a non-malperfusion patient [22]. This result is also attributable to selection bias, as patients with mesenteric malperfusion who recovered from endovascular reperfusion and subsequently underwent open aortic repair are a highly selected group. Patients within this group, who had intractable bowel ischemia were not progressed to central repair [22].

The timing of surgery is a contentious issue. Some studies advocate for normalisation of lactate, base excess prior to definitive surgery, ranging from 4 days to 2 weeks [7, 20, 22]. This delay needs to be balanced with the risk of aortic rupture, which *Deeb et al.* noted to be 5% per day [7]. Yang et al. advocated waiting two to four days [22]. Firstly, it enables the treating team to assess if the patient has a completely non-viable bowel, in which case they are not offered further aortic surgery as they have an unacceptably poor post-operative outcome [22]. Secondly, it allows circulating inflammatory mediators to clear, thus reducing the risk of postoperative multiorgan failure [7, 22]. Surgery was offered once PH was normalising and lactate was trending down [7, 22]. These studies however report a higher rate of aortic rupture in the interval period, although all aortic events in these studies occurred during earlier time periods when impulse control therapy was less commonly adhered to [7, 22]. Other studies performed central repair either immediately after or 24 H after mesenteric reperfusion. A low rate of rupture was noted in these studies, however could be associated with a higher post-operative mortality following central repair [18]. Based on the evidence, we would advocate for a delay of 48 to 72 H after mesenteric reperfusion, to enable the surgeon to assess the viability of the bowel, resuscitation in ICU and correct metabolic acidosis. This achieves a balance between a lower rate of rupture in select patients and minimising mortality following aortic repair.

The reperfusion strategy varied significantly across the studies. Two studies opted to proceed straight to laparotomy with stenting or anatomical bypass [19 21]. Laparotomy allows for assessment of bowel and definitive management of static obstruction, however subjects the patient to delay and morbidity. This treatment paradigm was confined to hospitals without access to hybrid operating suites. One study performed a TEVAR or axillary bifemoral bypass [18]. TEVARs may be fraught with issues. Firstly, the tissue in a dissected aorta is of poor quality and both the proximal and distal landing zones are not stable [20]. Secondly, they may cover up multiple intercostals increasing the risk of spinal ischemia [20]. The majority of studies performed fenestration as a first line intervention in the to correct dynamic obstruction, and this can be achieved within an hour of presentation [22]. Branch vessels may be further assessed for static obstruction (signified by an ongoing gradient across the vessel), in which case further intervention can take place. In instances of thrombus, a thrombectomy or local fibrinolysis can be performed [22]. This has the drawback of requiring access to an interventional or hybrid suite, which may not always be available [23].

Of note, however, is the selection bias present in this study. Patients who are too sick to undergo mesenteric reperfusion first undergo central repair, and make up the majority of the control cohorts. These patients are inherently more unwell and will have a higher mortality rate. Other studies introduce selection bias, as they stratified cohorts based on operative strategy which changed over time periods [7, 21]. Patients from earlier time periods largely underwent central repair first, whereas patients recruited in earlier time periods underwent delayed surgery. The mortality rate of ATAAD has decreased significantly over the past few decades which is a contributor to the bias in these studies. Other issues include small patient numbers the retrospective nature of studies. The ROBINS-I tool demonstrated this as the majority of evidence had serious risk of bias with only two studies deemed “moderate”. Further high-quality evidence, either randomised or propensity matched would be better poised to address this issue.

## Conclusion

ATAAD with mesenteric malperfusion is associated with high mortality and morbidity. Alternative treatment paradigms advocate for a mesenteric reperfusion first strategy, following then by delayed aortic surgery in the stable subset of patients. A summary of this evidence reveals a lower short-term mortality in hemodynamically stable patients with mesenteric malperfusion, along with an associated reduction in postoperative laparotomy/bowel resections. Of note, acute aortic events while awaiting definitive surgery were rare and were confined to one study in an early time period. Of those patients who survive to receive central repair, short-term mortality remains very low in the select group of hemodynamically stable patients. Further high-quality studies with randomized or propensitymatched data are required to verify these results.

### Electronic Supplementary Material

Below is the link to the electronic supplementary material.

Additional file Fig. 1: PRISMA flow-chart summarizing the search strategy for relevant publication.

Additional file Fig. 2: Risk Of Bias in Non-Randomized Studies of interventions (ROBINS-I) visual tool.

Additional file Fig. 3: leave one out analysis.

Additional file Fig. 4: Effect of study age on reported mortality.

## Data Availability

The datasets used and/or analysed during the current study are available from the corresponding author on reasonable request.
