# Correction: The role of delayed aortic surgery in type A aortic dissection and mesenteric ischemia: a systematic review and meta-analysis

**DOI:** 10.1186/s13019-023-02364-5

**Published:** 2023-09-15

**Authors:** Aditya Eranki, Ashley R Wilson-Smith, Michael L Williams, Aashray Gupta, Campbell Flynn, Jim Iliopoulos, Con Manganas

**Affiliations:** 1 Department of Cardiothoracic Surgery, Royal Prince Alfred Hospital, Sydney, Australia; 2 Department of Cardiothoracic Surgery, St George Hospital, Kograh, Sydney, 2217 Australia; 3 John Hunter Hospital, New Lambton Heights, Newcastle, Australia; 4 The Collaborative Research Group (CORE), Sydney, Australia; 5 The University of Sydney, Sydney, Australia; 6 Department of Cardiothoracic Surgery, Gold Coast University Hospital, Gold Coast, Australia; 7 University of Adelaide, Adelaide, Australia; 8 Department of Vascular Surgery, St George Hospital, Kograh, Sydney, Australia

Following publication of the original article [1], in this article the author name “Aashray Gupta” was incorrectly written as “Ashrey Gupta”.

The original article has been corrected.

## References

[1] Eranki, A., Wilson-Smith, A.R., Williams, M.L. et al. The role of delayed aortic surgery in type A aortic dissection and mesenteric ischemia: a systematic review and meta-analysis. J Cardiothorac Surg. 2023;18:247. 10.1186/s13019-023-02341-y. 10.1186/s13019-023-02341-y PMC10439544 37596605

