# Supplementary material for: The role of delayed aortic surgery in type A aortic dissection and mesenteric ischemia: a systematic review and meta-analysis
Source: J Cardiothorac Surg. 2023 Aug 18;18:247. doi: 10.1186/s13019-023-02341-y (PMC10439544; doi:10.1186/s13019-023-02341-y)
Supplement: Supplementary file 1 — Additional file Fig. 1: PRISMA flow-chart summarizing the search strategy for relevant publication. [file 13019_2023_2341_MOESM1_ESM.docx]

**Identification of studies via databases and registers**

Records removed *before screening*:

Duplicate records removed (n = 140)

Records identified from*:

Databases (n = 850)

Reference checks (n = 4)

Registers (n = 0)

**Identification**

Records screened

(n = 710)

Records excluded**

(n = 685)

Reports sought for retrieval

(n = 25)

Reports not retrieved

(n = 0)

**Screening**

Reports assessed for eligibility

(n = 25)

Reports excluded: 17

Wrong Study Design (n = 0)

Overlapping database (n = 3)

Abstract only (n = 1)

Does not differentiate Type A/Type B (n =1)

Studies included in review

(n = 8)

**Included**

**Supplementary Figure 1.** PRISMA flow-chart summarizing the search strategy for relevant publication
