# Supplementary material for: The role of delayed aortic surgery in type A aortic dissection and mesenteric ischemia: a systematic review and meta-analysis
Source: J Cardiothorac Surg. 2023 Aug 18;18:247. doi: 10.1186/s13019-023-02341-y (PMC10439544; doi:10.1186/s13019-023-02341-y)
Supplement: Supplementary file 4 — Additional file Fig. 4: Effect of study age on reported mortality. [file 13019_2023_2341_MOESM4_ESM.docx]

**Supplementary Figure 4: Effect of study age on reported mortality**
